# GPER1: A Hidden Key in Cardio-Renal Axis-Unveiling Its Therapeutic Potential for Heart and Kidney Disease

**DOI:** 10.1159/crm/abtag006

**Published:** 2026-09-07

**Authors:** Chenxi Wang, Sujie Guo, Yaodong Gu, Ye Cheng, Min Chen, Xiangchen Gu

**Affiliations:** Department of Nephrology, Yueyang Hospital of Integrated Traditional Chinese and Western Medicine, Shanghai University of Traditional Chinese Medicine, Shanghai, China

**Keywords:** Cardiovascular diseases, Kidney diseases, GPER1, G-1

## Abstract

**Background:**

Cardiovascular and kidney diseases are tightly interconnected through a bidirectional cardio-renal axis and exhibit pronounced sex-specific differences, implicating estrogen-associated signaling networks as critical modulators of disease susceptibility and progression. Among these, G protein-coupled estrogen receptor 1 (GPER1, GPR30) has emerged as a rapid signaling hub integrating vascular function, inflammatory responses, oxidative stress, and tissue remodeling across the cardiac, renal, and vascular tissues. However, interpretation of the extensive phenotype literature – largely derived from studies using the prototypical agonist G-1 – has become increasingly complex in light of recent advances in receptor structural biology and evolving physiological models.

**Summary:**

Accumulating evidence indicates that GPER1-associated signaling can attenuate maladaptive cardiac remodeling and inflammatory injury, modulate renal tubular transport and redox homeostasis, and preserve endothelial integrity through coordinated regulation of nitric oxide and endothelin pathways. Across organ systems, these effects converge on shared downstream signaling modules, including TGF-β/Smad, PI3K/Akt/eNOS, and NF-κB pathways. At the same time, emerging structural data, including identification of structurally supported agonists and alternative physiological activation mechanisms, challenge assumptions regarding direct ligand-receptor engagement. In this context, translational progress will require a more rigorous framework that integrates structure-guided pharmacology, standardized validation linking ligand binding to downstream signaling in matched experimental systems, and genetic epistasis approaches combining loss-of-function and rescue strategies.

**Key Messages:**

Rigorous mechanistic validation is essential to establish causal GPER1-associated signaling across the cardio-renal axis. Such approaches are required to determine whether GPER1 represents a tractable therapeutic target in combined cardiac and kidney disease.

## Introduction

### Interplay between Cardiovascular and Kidney Diseases

Cardiovascular diseases (CVDs) and kidney diseases are major public health challenges that frequently co-occur and interact through shared pathophysiological mechanisms, including inflammation, fibrosis, endothelial dysfunction, and oxidative stress [1]. Chronic kidney disease (CKD) and CVD often form a self-reinforcing cycle in which renal injury promotes cardiovascular (CV) remodeling and events, while CV dysfunction worsens renal perfusion and accelerates renal damage [1]. Substantial evidence [2] indicates that CKD markedly increases the risk of adverse CV outcomes, including heart failure (HF), myocardial infarction (MI), and CV death [3]. HF, in particular, is a major contributor to mortality in the CKD population [4]. Conversely, across CKD stages, CV complications remain the leading cause of death [5–7]. CV abnormalities such as hypertension and HF can cause renal hypoperfusion, ischemic injury, neurohormonal activation, and progressive loss of nephron mass, ultimately driving further decline in renal function [8].

Clinical indicators of impaired renal function- including estimated glomerular filtration rate (eGFR), urinary albumin-to-creatinine ratio (UACR), and blood urea nitrogen (BUN), are robust predictors of CV events and mortality [9, 10]. Importantly, even modest reductions in eGFR or mild elevations in UACR and blood urea nitrogen are associated with meaningful increases in CV events and all-cause mortality, highlighting their prognostic significance and their association with systemic vascular dysfunction [5, 11, 12]. Mechanistically, chronic inflammation contributes to vascular and renal damage by inducing endothelial activation, immune cell recruitment and tissue remodeling programs [13]. Fibrosis, often sustained by persistent inflammatory signaling and maladaptive repair, disrupts organ architecture and impairs function in both myocardium and kidneys [14, 15]. Endothelial dysfunction further amplifies pathology by promoting abnormal vascular permeability, thrombosis, and impaired tissue perfusion-core processes that drive cardio-renal disease progression [16, 17].

The heart and kidneys maintain CV homeostasis through multiple interfaces, including hemodynamics, neurohormonal activity, and biochemical metabolism. Their functions are interdependent, and acute or chronic dysfunction in one organ can readily induce injury in the other – a relationship particularly exemplified by cardiorenal syndrome [18]. The interactions between the heart and kidneys span multiple pathological processes – including hemodynamic abnormalities, atherosclerosis, neurohormonal activation, inflammation, and disturbances in the anemia-bone-mineral axis – forming the complex and tightly intertwined clinical and pathophysiological foundation of cardiorenal syndrome [19]. Given this interdependence, therapeutic strategies that target shared mechanisms across the cardio-renal axis are increasingly recognized as essential for improving clinical outcomes.

### Estrogen-Associated Signaling and Cardio-Renal Integration

There are significant sex differences in the prevalence, clinical manifestations, disease progression, and prognosis of CVD and kidney disease. The mechanism underlying these differences is complex, which is closely related to various factors such as metabolic stress response, sex hormone regulation, sex chromosome differences, and accessibility of sex-related risk-modulating therapies [20, 21].

Epidemiological studies consistently demonstrate sex differences in the prevalence, clinical presentation, and outcomes of both CV and kidney diseases, suggesting sex hormones and their downstream signaling networks contribute to disease susceptibility and progression [22]. In CV, renal, and immune systems, estrogen-associated signaling has been linked to anti-inflammatory and anti-apoptotic actions, as cytoprotection, motivating continued interest in estrogen-responsive receptors as potential modulators of cardio-renal disease biology [22, 23].

### Brief Introduction to GPER1 (GPR30)

G protein-coupled receptors constitute the largest family of membrane receptors and regulate many aspects of CV and renal physiology, including hemodynamic control, fluid-electrolyte balance and inflammatory signaling [24]. Within this superfamily, G protein-coupled estrogen receptor 1 (GPER1) has emerged as a candidate mediator of rapid estrogen-related signaling. GPER1 is a seven-transmembrane receptor whose reported subcellular localization may vary by cell types and experimental conditions, with studies describing distribution at the cellular membrane as well as intracellular membranes such as endoplasmic reticulum and mitochondria [25]. GPER1 has been reported to be expressed across diverse tissues and cell types, including cardiac and renal cell populations as well as immune cells, suggesting that it may participate in multi-organ regulation rather than acting in a single compartment [26]. Functionally, GPER1 has been linked to coupling with multiple G protein α-subunits (Gαs, Gαi/o, Gαq/11, and Gα12/13), implying that it can engage distinct downstream signaling pathways depending on cellular context and ligand condition [27]. These properties make GPER1 conceptually well-suited to influence integrated physiological processes that bridge the CV and renal systems.

In the context of the cardio-renal axis, GPER1 has therefore attracted interest as a potential regulator of homeostasis and injury responses. Rather than focusing on a single organ, an emerging theme is that GPER1-associated signaling as shown in Figure 1 may participate in coordinated control of (i) tissue remodeling regulatory pathways; (ii)functional adaptation to stress; (iii) vascular/endothelial signaling that shapes organ perfusion and systemic hemodynamics; and (iv) oxidative and inflammatory pathways that drive progressive damage across organs [28]. However, the mechanistic interpretation of these findings remains complicated. While the tool compound G-1 has been widely used to define GPER1 biology, recent structural evidence challenges its direct binding to the receptor, suggesting that the reported cardio-renal protective effects may involve alternative mechanisms or complex conformational states (as critically discussed in The Controversy over G-1 and Target Engagement of GPER1).

**Fig. 1.**
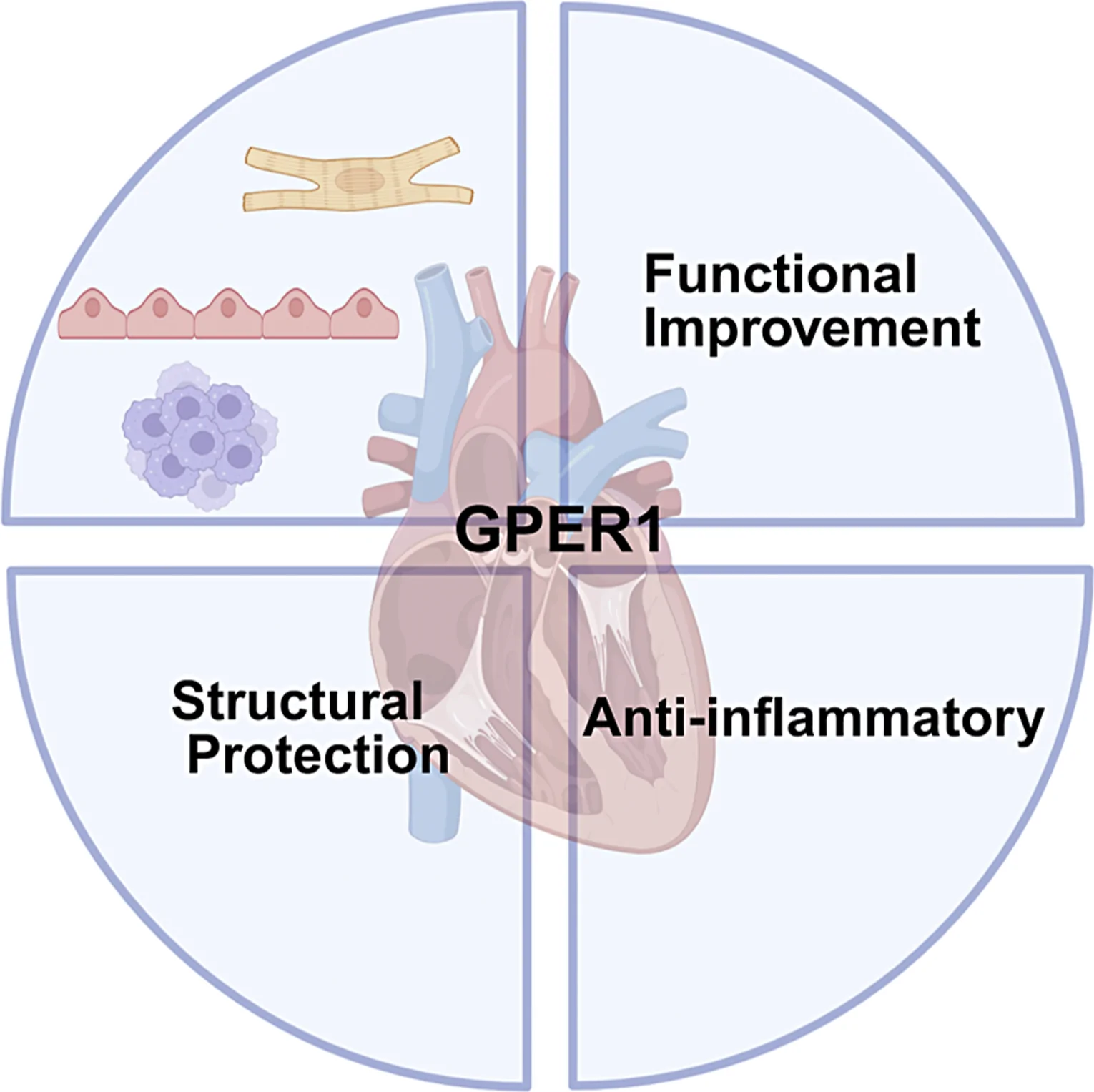
Cardiac effects of GPER1.Created in BioRender.

It is important to note that while recent structural evidence has challenged the direct binding of G-1 to GPER1, the extensive phenotypic data accumulated over the past decade remain biologically informative [29, 30]. These findings consistently point to the existence of a “GPER1-associated signaling” network or downstream effector pathways that are accessible via the G-1 chemical scaffold [31–34]. Therefore, in this review, we synthesize these functional studies to highlight the therapeutic potential of this signaling axis, while acknowledging that the precise molecular engagement – whether direct or indirect – is currently being redefined.

This review aims to systematically characterize the regulatory roles, molecular mechanisms, and translational value of GPER1 in the pathological progression of CV and kidney diseases. To achieve this, we conducted a comprehensive, unrestricted time-range search of the latest peer-reviewed published literature in the PubMed database, using heart, cardiac, CV, kidney, renal, GPER1, and GPR30 as the core search keywords.

## Cardiac Effects

GPER1 is expressed across multiple cardiac-relevant cell types, including cardiomyocytes, endothelial cells, and immune cells such as macrophages [35]. Collectively, as shown in Figure 1, available evidence suggests that activation of GPER1-associated signaling can confer cardioprotection by coordinately modulating structural remodeling, contractile function, and inflammatory pathways, although the magnitude and direction of these effects may vary across models and biological context [24, 36–49].

Notably, cardiac GPER1 expression has been reported to exhibit sex-related differences, which may contribute to differential susceptibility to pressure overload-induced remodeling; female hearts are often more resistant to hypertrophic stress than male hearts [46]. Together, these observations position GPER1 as a plausible mediator linking sex-related biology to myocardial adaptation and injury responses.

### Structural Protection

Activation of GPER1-associated signaling has been repeatedly linked to structural cardioprotection, including reduced cardiac fibrosis [24, 36–40], attenuated hypertrophic remodeling [24, 47, 48], smaller infarct size [37], and lower oxidative stress across diverse preclinical models [37]. Genetic evidence supports a causal contribution, as cardiomyocyte-specific Gper1 knockout models exhibited impaired structural and functional resilience under stress [41]. Mechanistically, reported anti-fibrotic effects converge on inhibition of canonical profibrotic signaling, including upregulation of Smad7 and consequent suppression of the TGF-β/Smad activity [42]. In parallel, stromal remodeling may be restrained by limiting cardiac fibroblast expansion (via downregulation of cell-cycle regulators) [43] and by reducing extracellular matrix-remodeling signals such as ERK-dependent MMP-9 signaling, with downstream suppression of TGF-β1-linked responses [40]. Taken together, these data support a model in which GPER1-associated signaling attenuates maladaptive myocardial remodeling through coordinated suppression of profibrotic signaling and fibroblast expansion [24, 36–43, 49].

### Functional Improvement and Cardioprotection

Beyond remodeling, GPER1-associated signaling has been linked to functional benefits in cardiac performance and hemodynamic regulation. Across several models, pharmacological stimulation has been associated with lower blood pressure and improved cardiac function, consistent with a combined vascular-cardiac mechanism involving pathways related to aldosterone and endothelin signaling [38, 49]. At cardiomyocyte level, GPER1 has been implicated in rapid signaling that shapes excitation-contraction coupling, Ca2+ handling, and stress responsiveness, including modulation of β2-adrenergic signaling balance [33, 50, 51]. In ischemia-reperfusion injury and HF models, cardioprotection has been linked to activation of PI3K/AKT signaling and mitochondrial protection via Sirt1/3/p-AMPK/UCP2 pathways [52, 53], consistent with preservation of bioenergetic capacity under stress. GPER1 activation reduces Epi-elicited mortality and cardiac damage through mechanisms involving its downstream Gαs and Gαi pathways [54]. GPER1 adaptively regulates the Gαi/cAMP ratio [51], and mediates estrogen’s cardioprotective effects by balancing Gαs/Gαi signaling activation under stress [55]. Although the magnitude and direction of effects can vary by sex, model, and experimental system, the aggregate literature [44, 45, 56–59] supports GPER1-associated signaling as a multifunctional axis capable of improving contractile performance through both systemic hemodynamics and cardiomyocyte-intrinsic stress adaptation. Overall, current evidence supports GPER1-associated signaling as a multifunctional regulator of cardiac physiology, acting through both cardiomyocyte-intrinsic mechanism and hemodynamic pathways.

### Anti-Inflammatory Actions

GPER1 is increasingly recognized as a regulator of cardiac inflammatory responses. Loss of GPER1-associated signaling promotes inflammatory hyperactivation and exacerbates tissue injury across multiple experimental contexts [36, 60–65], supporting an endogenous protective role. Consistent with this, cardiomyocyte-specific GPER1 deletion induces sex-dependent adverse remodeling, accompanied by coordinated alterations in mitochondrial inflammatory gene expression [66]; transcriptomic enrichment analyses further highlight systemic shifts in these pathways, supporting a transcriptional basis for GPER1-mediated control of the inflammatory microenvironment during cardiac injury [66]. In sepsis models, pharmacological stimulation has been reported to improve cardiac function while reducing oxidative stress and apoptosis, potentially involving STAT6/ACE2/c-fos signaling and PPARδ activation [67, 68]. The NLRP3 inflammasome has also been proposed as a downstream node: cardiomyocyte-specific Gper1 deletion is accompanied by inflammasome-related transcriptional activation and NLRP3 inhibition attenuates hypertrophic remodeling and improves functional roles in vivo [67, 69]. Together, these findings support the concept that GPER1-associated signaling couples mitochondrial homeostasis to suppression of maladaptive inflammatory amplification in the injured heart.

## Renal Effects

GPER1 is widely distributed within the kidney and has been reported in multiple renal-relevant cell types including tubular epithelial cells, podocytes, endothelial cells, and macrophages. Across models of acute and chronic renal injury, as shown in Figure 2, renal actions attributed to GPER1-associated signaling can be divided into three interrelated domains: (i) structural remodeling (fibrosis), (ii) inflammatory and immune regulation (including macrophages), and (iii) renal physiology encompassing hemodynamics/perfusion, tubular transport, and redox homeostasis.

**Fig. 2.**
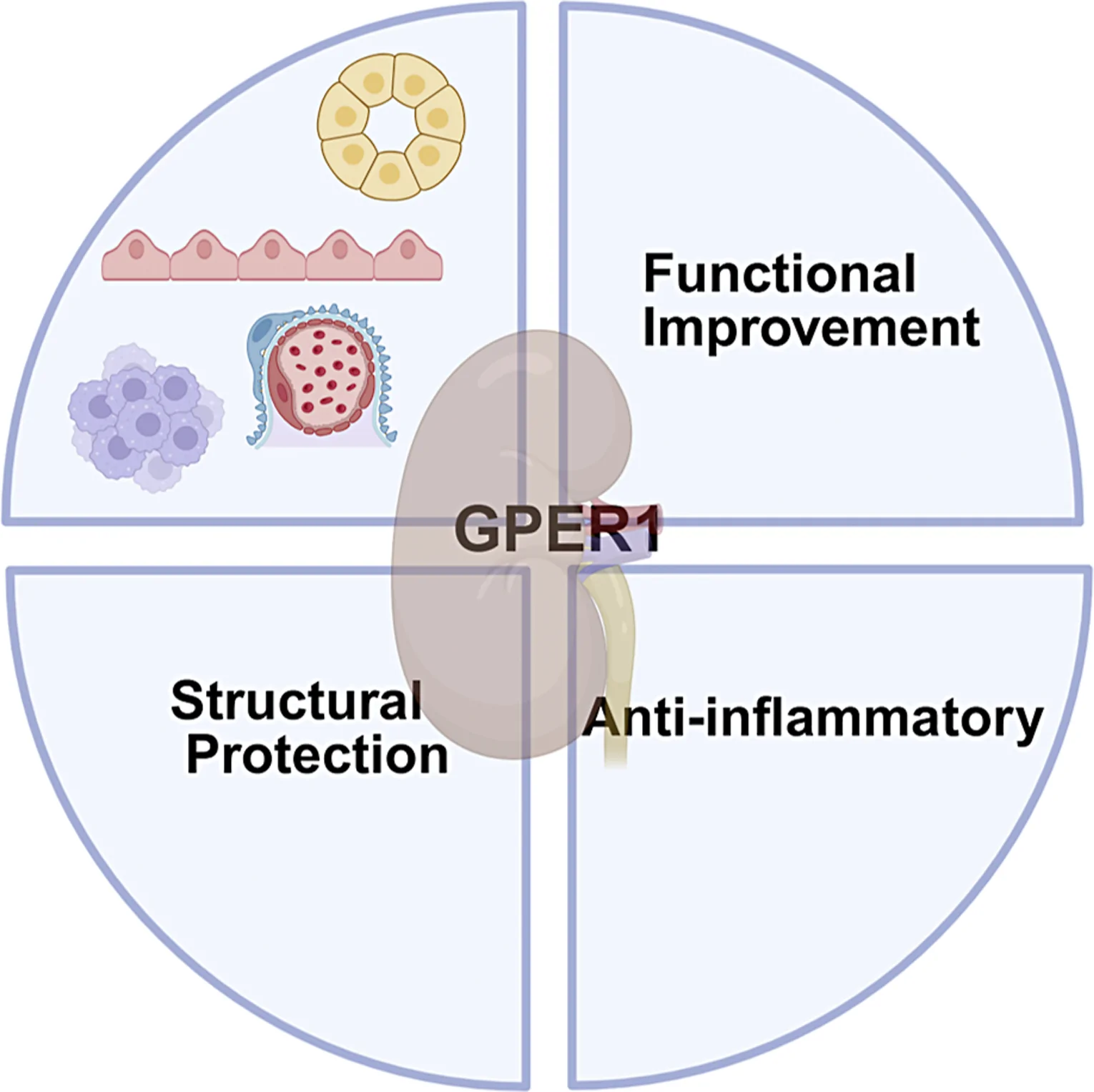
Renal effects of GPER1.Created in BioRender.

### Structural Remodeling: Anti-Fibrotic Effects

GPER1 activation has been associated with anti-fibrotic effects in experimental kidney disease [70]. In chronic injury settings, including cisplatin-associated and obstructive phenotypes, pharmacological activation of GPER1 has been reported to attenuate renal fibrosis and maladaptive remodeling [36, 71]. Conversely, pharmacologic blockade of GPER1 exacerbates fibrotic progression in the unilateral ureteral obstruction (UUO) model [72]. A key mechanistic axis involves the suppression of macrophage-driven fibrogenesis, including restraint of both M1 and M2 macrophage polarization [36]. Together, these findings support GPER1 as a regulator of inflammatory-fibrotic coupling that sustains extracellular matrix accumulation and chronic renal remodeling.

### Anti-Inflammatory and Immunomodulatory Effects

Beyond fibrosis, GPER1 is increasingly recognized as a modulator of renal inflammation in obstructive and chronic injury contexts. Consistent with an endogenous protective role, Gper1 deficiency intensifies inflammatory activation and aggravates downstream fibrotic responses [36]. Conversely, pharmacological activation using G-1 has been reported to shift immune responses toward an anti-inflammatory profile, characterized by increased IL-10 and reduced Th17 expression, regulated via TGF-β signaling [71]. In vitro evidence suggests that these effects may involve downregulation of immune-related pathways and inhibition of MAPK pathway [36]. In addition, certain bioactive compounds, including components derived from Achyranthes, have been reported to alleviate kidney injury via GPER1-dependent mechanisms [73]. Collectively, these studies support the concept that GPER1 can shape the renal immune microenvironment, thereby influencing downstream remodeling trajectories.

### Renal Physiology and Functional Outcomes: Perfusion, Redox Homeostasis, and Tubular Transport

GPER1-associated signaling has also been linked to preservation of renal function and improved renal perfusion [74]. Mechanistically, activation of this pathway has been reported to enhance antioxidant defenses via the Nrf2 axis and its downstream enzymes, thereby limiting oxidative stress that contributes to endothelial dysfunction and tubular injury [71]. Podocyte-protective effects have also been described; for example, Icariin has been reported to stabilize podocytes through a GPER1-associated mechanism by promoting the mitochondrial Bcl-2 translocation and reducing downstream apoptosis [75]. In parallel, kidney GPER1-associated signaling influences tubular sodium transport, including modulation of epithelial sodium channel activity and promotion of natriuresis, with stronger effects reported in females [76]. Consistent with this sex-dependent physiology, pharmacological stimulation with G-1 promotes natriuresis and diuresis in female rats but not in males [77]. Complementing the epithelial sodium channel axis, GPER1 has also been reported to upregulate the kidney Cx30/ATP/P2Y2 purinergic signaling pathway, which may contribute to blood pressure lowering response associated with GPER1 activation [78].

Notably, the regulation of natriuresis and blood pressure by GPER1 exhibits significant sex dimorphism, often showing more pronounced protective effects in females. This observation holds potential translational significance for precision medicine in hypertension and CKD. If GPER1 signaling proves to be a predominant regulatory mechanism in the female renal physiology, it suggests that female patients might require distinct therapeutic strategies compared to males. Consequently, future translational efforts should prioritize sex-stratified approaches to determine whether targeting the GPER1 axis offers superior efficacy for women with hypertensive kidney disease, moving beyond a “one-size-fits-all” treatment paradigm. Taken together, these findings support a framework in which GPER1 influences renal outcomes through integrated regulation of renal hemodynamics (perfusion), tubular transport (natriuresis/diuresis), oxidative stress defenses (Nrf2), and – over longer time scales – structural remodeling, thereby linking early functional preservation with subsequent tissue protection.

## Vasculature

GPER1 exerts protective actions within the vasculature and contributes to vascular homeostasis and blood pressure regulation across organ systems. GPER1 plays a significant regulatory role in the pathophysiological functions of endothelial cells and vascular smooth muscle cells [79, 80]. In the CV system, GPER1 is expressed in vascular endothelial cells, where it has been reported to mitigate atherosclerosis [35] and contributes to blood pressure control [38, 49, 81]. Within the renal vasculature, GPER1 modulates vascular responses through signaling pathways that include ROCK, PKC, and MAPKs [82], supporting a nephroprotective effect in salt-induced hypertension models [83]. GPER1 has also been described as a sex-specific upstream regulator of the renal endothelin-1 (ET-1) system [84]. Consistent with these mechanisms, G-1 promotes vasodilation by increasing nitric oxide (NO) bioavailability while reducing ET-1-associated signaling [71]. In addition, G-1 has been reported to prevent oscillatory shear stress-induced oxidative stress, inflammatory cytokines, and adhesion molecule expression, supporting a direct endothelial-protective profile relevant to systemic vascular injury [35].

Critically, pharmacological activation of this pathway has been shown to reverse maladaptive stiffening. Treatment with LNS8801, a novel, enantiomerically pure agonist derived from the G-1 scaffold, was found to be more effective than the racemic G-1 mixture in reversing ovariectomy-induced arterial stiffness [85]. LNS8801 treatment restored the expression of contractile genes and reduced pulse wave velocity (PWV) to pre-ovariectomy levels without altering vascular geometry [85]. While these data strongly suggest that the G-1/LNS8801 chemical scaffold confers vascular protection, the precise molecular target of these compounds – whether GPER1 or an alternative sensor – is currently being re-evaluated in light of recent structural evidence (as discussed in The Controversy over G-1 and Target Engagement of GPER1) [85]. Overall, as shown in Figure 3**,** vascular/endothelial mechanisms provide a plausible system-level bridge by which GPER1 could coordinately influence cardiac load, renal perfusion, and the progression of cardio-renal disease.

**Fig. 3.**
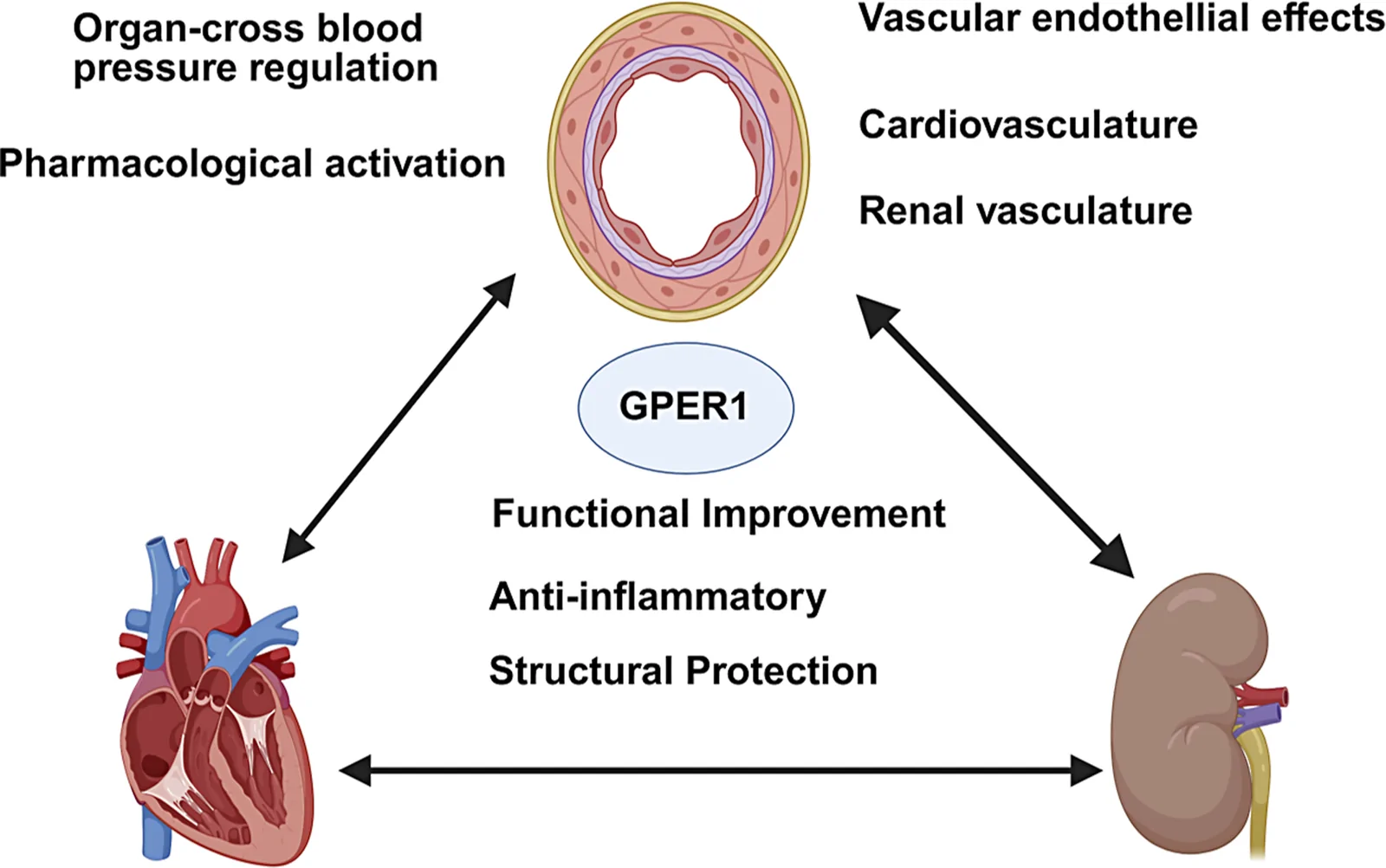
GPER1-associated function in the cardio-renal axis. GPER1 functions as an integrative signaling hub linking cardiac, renal, and vascular protection through functional improvement, anti-inflammatory and structural protection. Referenced and modified from Chen 2021 [35], Xie 2023 [36], Lai 2025 [38], Liu 2022 [42], Chang 2019 [70], and Ahmed 2024 [71]. Created in BioRender.

## Convergent Pathways in the Cardio-Renal Axis

A central concept emerging from organ-specific studies is that GPER1 may confer coordinated protection in both heart and kidneys by regulating shared signaling nodes. Current evidence highlights three major pathway modules that recur across tissues: TGF-β/Smad, PI3K/Akt/eNOS, and NF-κB [42, 53, 86–89]. Within the TGF-β/Smad axis, GPER1 activation inhibits profibrotic signal transduction in both organs. In the heart, suppression of this pathway is associated with reduced atrial fibrosis and decreased risk of atrial fibrillation [42]. Within the PI3K/Akt/eNOS pathway, GPER1 activation improves endothelial function and suppresses oxidative stress across both cardiac and kidney diseases [90, 91]. In the heart, this pathway contributes to reduced myocardial infarct size and attenuation of pathological hypertrophy [53, 88]. In the renal tissues, PI3K/Akt/eNOS signaling has also been linked to sodium balance regulation and blood pressure homeostasis, supporting an integrated hemodynamic and tubular transport role [92]. Within the NF-κB pathway, GPER1 signaling consistently suppresses inflammatory activation, thereby alleviating inflammatory responses in both cardiac and renal tissues [93, 94].

In summary, as shown in Figure 4 and Table 1**,** through these convergent pathways, GPER1 may deliver multi-dimensional protection – anti-fibrotic, endothelial-stabilizing, antioxidant, and anti-inflammatory – supporting its potential relevance as a therapeutic target for cardiorenal diseases.

**Fig. 4.**
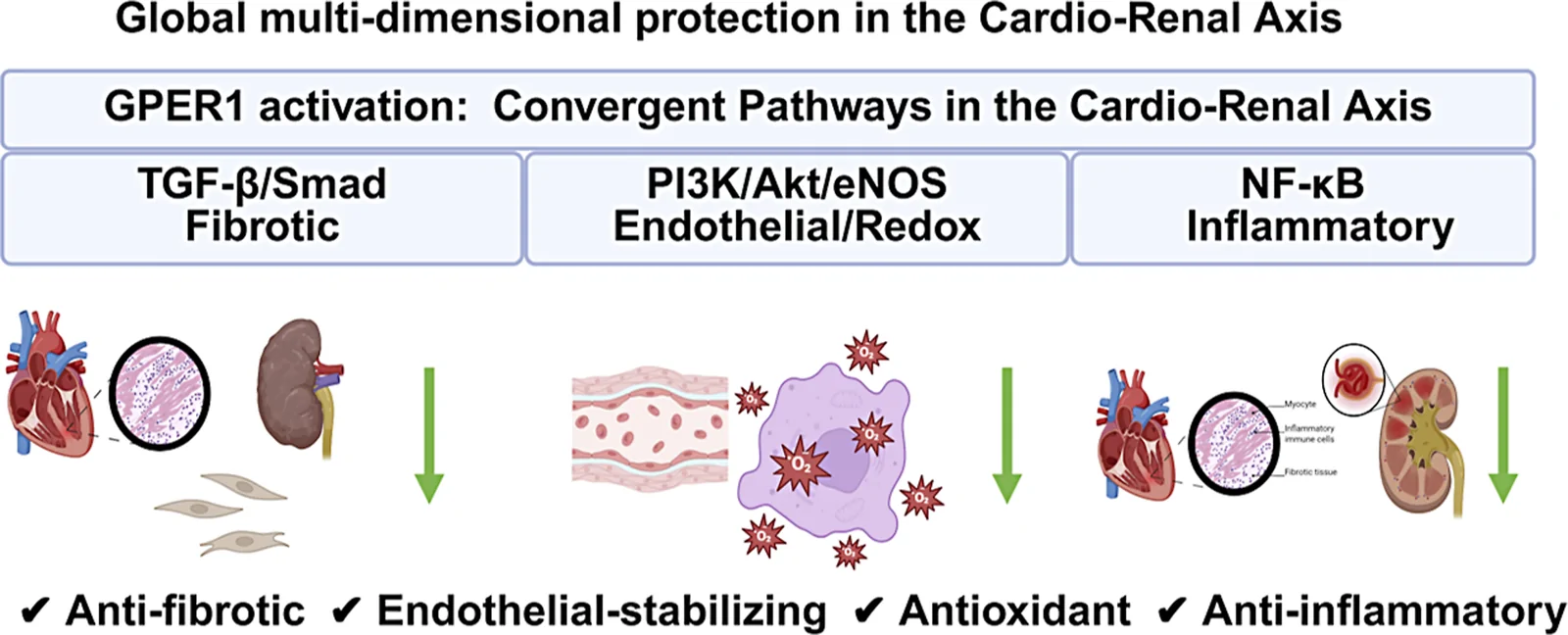
GPER1-associated signaling in the cardio-renal axis. GPER1 functions as an integrative signaling hub linking cardiac, renal, and vascular protection through convergent regulation of profibrotic, endothelial/redox, and inflammatory pathways. The schematic highlights anti-fibrotic, endothelial-stabilizing, antioxidant, and anti-inflammatory effects across organs. Referenced and modified from Liu 2022 [42], Wang 2019 [53], Li 2014 [86], Chakrabarti 2012 [87], Pei 2019 [88], De Giusti 2015 [89] and Lee 2014 [90]. Created in BioRender.

**Table 1.** The diverse effects of GPER1 across organs and cell types

Organ system	Key cell types expressed	Core biological functions of GPER1-associated signaling
Heart	Cardiomyocytes, endothelial cells, macrophages	1.Structural protection: attenuate cardiac fibrosis/hypertrophic remodeling, reduce infarct size
		2.Functional improvement: enhance cardiac performance, regulate hemodynamics, lower blood pressure
		3.Anti-inflammation: suppress maladaptive inflammatory activation, mitigate oxidative stress
Kidney	Tubular epithelial cells, podocytes, endothelial cells, macrophages	1.Structural protection: attenuate renal chronic injury-induced fibrosis and maladaptive remodeling
		2.Functional preservation: improve renal perfusion, maintain redox homeostasis, regulate tubular transport, protect podocytes
		3.Anti-inflammation: shift immune responses to anti-inflammatory profile, suppress macrophage polarization
Vasculature	Endothelial cells, vascular smooth muscle cells	1. Vascular homeostasis: mitigate atherosclerosis, regulate blood pressure
		2. Endothelial protection: enhance NO bioavailability (vasodilation), suppress ET-1 signaling, inhibit oxidative stress/inflammatory cytokine expression
		3. Anti-stiffening: reverse maladaptive vascular stiffening

## The Controversy over G-1 and Target Engagement of GPER1

In GPER1-targeted research, the most widely used pharmacological probe is G-1, commonly regarded as an agonist alongside G-15/G-36 [95, 96], frequently used as an antagonist [97]. However, an ongoing debate centers on whether G-1 directly activates GPER1 and specifically activates GPER1 in all experimental contexts. G-1 was first reported by Bologa et al. in 2006 following virtual screening and experimental characterization that included fluorescent estrogen-derivative binding assays, high-throughput flow cytometry (HyperCyt), and assays designed to capture estrogen-associated PI3K activation [95].

Since then, a substantial literature has used G-1 and described phenotypes consistent with activation of GPER1-linked pathways across CV, renal, vascular, and metabolic contexts [33, 34]. G1-induced selective GPER1 activation mediates non-genomic effects in hypertrophic hearts through reduced ICaL and increased potassium currents, with potential cardioprotective properties [33]. However, pharmacological responsiveness alone does not establish target engagement, and the breadth of G-1 responsive phenotypes across organs underscores the need for orthogonal validation when attributing effects specifically to GPER1. Importantly, the extensive disease-phenotype literature obtained with G-1 remains biologically informative, but its interpretation now requires careful separation of pharmacology from receptor attribution.

Notably, as shown in Figure 5, earlier pharmacological work reported high-affinity, competitive binding of 17β-estradiol to GPER1, supporting its historical designation as a transmembrane estrogen receptor. In contrast, several studies have directly challenged whether GPER1 binds estradiol or whether G-1 selectively activates GPER1 in standardized receptor assays. Otto C et al. reported that GPER1 did not bind estradiol or transduce signaling in response to it in their experimental systems, and that G-1 did not trigger classical estrogen-mediated responses in uterus or breast [31]; Tutzauer J et al. further argued GPER1 cannot be classified as an estrogen receptor, reporting no detectable G-1- or estradiol- stimulated activity across multiple in vitro receptor assay [32].

**Fig. 5.**
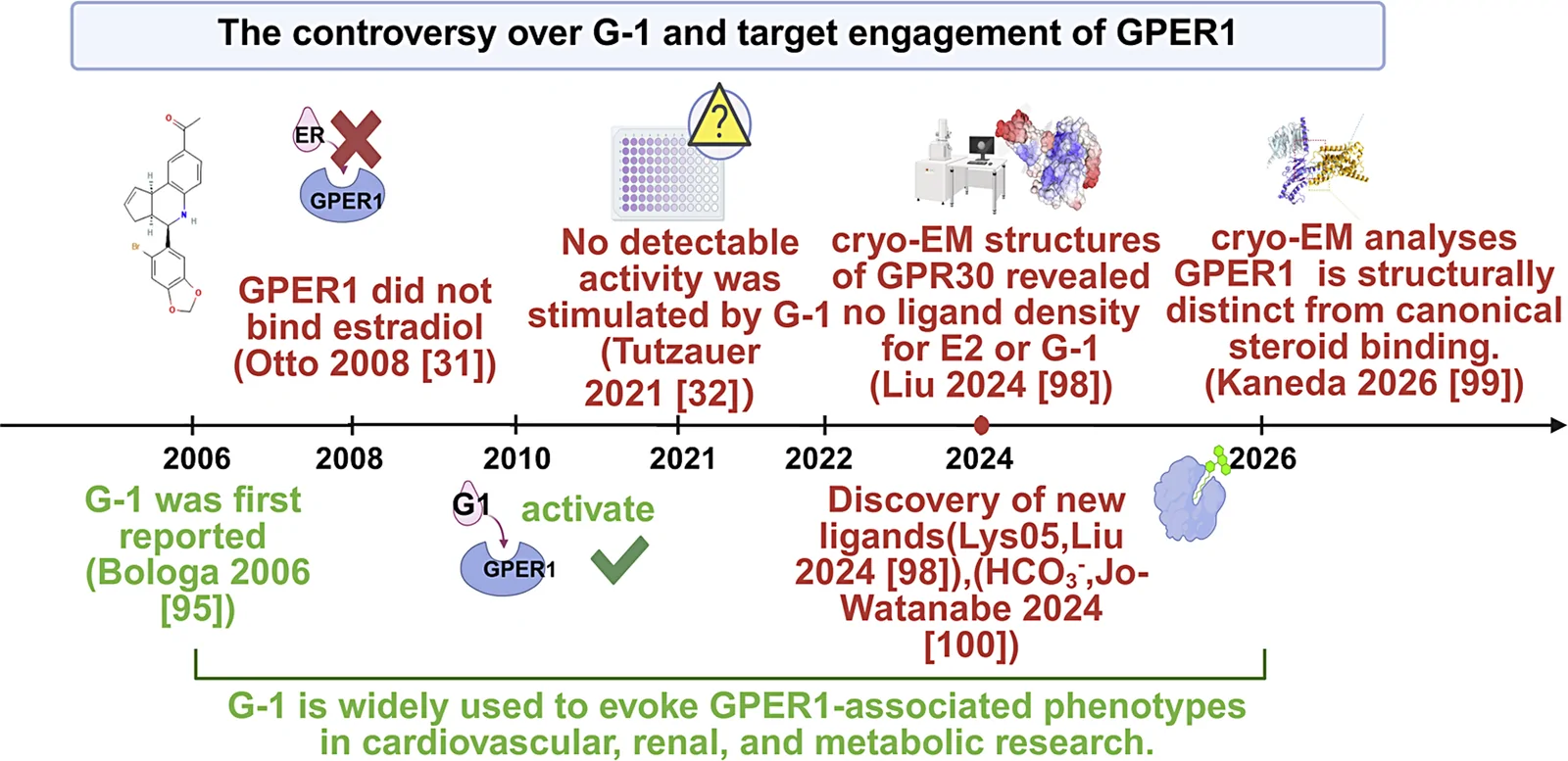
The controversy over G-1 and target engagement of GPER1.This panel summarizes the evolving re-interpretation of G-1 pharmacology, informed by recent cryo-EM and physiological studies that challenge direct receptor agonism and emphasize the need for structure-guided validation of GPER1 target engagement. Referenced and modified from Bologa 2006 [95], Otto 2008 [31], Tutzauer 2021 [32], Liu 2024 [98], Jo-Watanabe 2024 [99], and Kaneda 2026 [100]. Created in BioRender.

However, the historical classification of GPER1 as a direct estrogen receptor has faced its most significant challenge from structural biology in 2024. Liu et al. resolved the high-resolution cryo-EM structures of GPR30-Gq complexes and revealed an orthosteric pocket whose physicochemical properties are unfavorable for hydrophobic steroids such as estradiol; notably, no ligand density corresponding to E2 or G-1 was observed under the resolved conditions, motivating re-evaluation of direct binding [98]. Instead, the authors identified a positively charged molecule, Lys05, as a structurally supported agonist [98]. Complementary cryo-EM analyses further revealed a highly polar extracellular architecture comprising multiple charged pockets, with structure-guided mutagenesis and calcium-signaling assays supporting bicarbonate-dependent receptor activation despite the absence of directly resolved ligand density [100]. Collectively, these structural features are incompatible with canonical steroid binding and necessitate a clearer distinction between G-1-responsive phenotypes and bona fide GPER1 target engagement [100]. In parallel, Jo-Watanabe et al. provided compelling evidence identifying GPR30 as a physiological receptor for bicarbonate anions, acting in a pH-independent manner, thereby supporting an alternative physiological modality-bicarbonate sensing-that may operate alongside, or independently of, estrogen-associated signaling [99].

Together, these findings underscore the limitations of inferring GPER1 target engagement from G-1 phenotypes alone and highlight the need for validation. Collectively, these findings raise the possibility that at least a subset of reported G-1 effects may reflect context-dependent receptor states, indirect signaling mechanisms, or off-target activities, rather than uniform, direct agonism of GPER1.

## Prospective Outlook

Accumulating evidence over the past decade has linked GPER1 (GPR30) to regulatory signaling across the cardiovascular-renal continuum, including vascular homeostasis, endothelial function, and remodeling. However, recent structural and functional redefinition of GPER1 requires a reassessment of prior pharmacology-driven conclusions. The emerging identification of GPER1 as a bicarbonate sensor introduces a new physiological framework that is highly relevant to renal acid-base regulation and systemic homeostasis, and it raises the possibility that GPER1 couples disturbances such as CKD-associated metabolic acidosis to changes in vascular homeostasis, perfusion, and tissue remodeling.

Future priorities should therefore focus on: (i) re-validating key cardio-renal phenotypes using structurally supported agonists (e.g., Lys05) and/or controlled bicarbonate perturbations to establish bona fide target engagement; (ii) defining the true molecular mechanism and target(s) responsible for the extensive disease-phenotype literature attributed to G-1; and (iii) testing whether GPER1 signaling modulates renal hemodynamics, electrolyte transport, and ischemic injury responses specifically under dynamic acid-base conditions, thereby clarifying its role as a metabolic sensor within the cardio-renal axis.

## Conclusion

In summary, GPER1 remains a potentially important integrative node, but mechanistic clarity – combining structure-guided pharmacology with rigorous physiology – will be decisive for determining its translational value in heart and kidney disease.

## Conflict of Interest Statement

The authors declare that they do not have any conflict of interest.

## Funding Sources

This study was supported by the National Natural Science Foundation of China (Grant No. 82474175, to X. Gu) and the 2023 Shanghai “Science and Technology Innovation Action Plan” Biopharmaceutical Technology Support Special Project (Grant No. 23S11900500, to x. Gu). All funders only provided financial support for the research and were not involved in study design, experimental execution, data analysis, manuscript conception, planning, and writing-up or the decision for publication.

## Author Contributions

Writing – original draft: C.W.; editing: Y.G., S.G.,Y.C., and M.C.; conceptualization, writing – review and editing: X.G.
